# Predicting the winning team in basketball: A novel approach

**DOI:** 10.1016/j.heliyon.2022.e12189

**Published:** 2022-12-09

**Authors:** Cem Osken, Ceylan Onay

**Affiliations:** a Dept. of Management Information Systems, Bogazici University, Istanbul, Turkey; b School of Business and Law, University of Brighton, Elm House, Lewes Road, Brighton, BN2 4NU, United Kingdom

**Keywords:** Sports analytics, Basketball, Genetic algorithms, Artificial neural networks, Complex systems, Prototype heuristics

## Abstract

Predicting the winner of a basketball game is a difficult task, due to the inherent complexity of team sports. All 10 players on the court interact with each other and this intricate web of relationships makes the prediction task difficult, especially if the prediction model aims to account for how different players amplify or inhibit other players. Building our approach on complex systems and prototype heuristics, we identify player types through clustering and use cluster memberships to train prediction models. We achieve a prediction accuracy of ∼76% over a period of five NBA seasons and a prediction accuracy of ∼71% over a season not used for model training. Our best models outperform human experts on prediction accuracy. Our research contributes to the literature by showing that player stereotypes extracted from individual statistics are a valid approach to predict game winners.

## 1 Introduction

### 1.1 Overview

Basketball can be analysed using the complex systems theory: two teams, each with five players on the court and seven substitutes (five in the case of International Basketball Federation (FIBA) rules being used), in order to achieve a goal, acting on their training and intuition whilst also reacting to each other's actions and perceived intents (García et al., 2013). A similar approach is to think of sports teams as superorganisms, with complex feedback loops and integration capabilities (Duarte et al., 2012). The complexity of such structures yields a high level of uncertainty on the likelihood of achieving the objective (Martinez, 2017). How efficiently the components of a team interact to create synergies and adapt to opponents is not a trivial question to answer yet needs to be discussed to successfully predict game results. Therefore, we posit that understanding the players' roles and interactions is a foundational element of prediction exercises.

Predicting the outcome of a basketball game is of interest to many stakeholders: coaches and team managers so they can identify optimal team compositions, betting companies so they can model the odds for financial performance, casual fans purely for entertainment purposes are but a few (Schumaker et al., 2010) of these stakeholders. Nevertheless, this prediction problem is by no means a trivial one. How efficiently the components of a team interact to create synergies and adapt to opponents is not a trivial question to answer yet needs to be discussed to successfully predict game results.

Historically, a strict classification of player roles has been used to help simplify the interactions within basketball teams. It is worth noting that the game has been evolving, especially in the last two decades (Safir, 2015). Power forwards and centres, people who historically were confined to operate under the basket to leverage their height and physical advantages, now shoot three pointers and some even handle the ball distribution duties in their teams and have more assists per game than many point guards (due to their role as the primary distributor of the ball, PGs were historically players with highest per game assist figures by far). In-depth statistical analysis capabilities granted by player tracking, play-by-play data, and machine learning have led the teams to seek efficiency gains wherever they can be found, utilising synergies among certain line ups or exploiting certain weaknesses in opposing teams. This trend results in players transcending the boundaries of their positions (Rangel et al., 2019).

When the components themselves cannot be easily classified or modelled, understanding how complex systems operate becomes an even more difficult task. That being said, the relatively low number of agents (i.e., players) involved in sports allow us to model these environments (Passos et al., 2011) and understand how the agents interact to enhance the probability of achieving the outcome. Therefore, we posit that the traditional player positions need to be redefined in order to capture the tendencies of player archetypes which can then be used to understand how certain archetypes yield positive or negative synergies, impacting the likelihood of team success.

### 1.2 Problem statement and hypotheses

The exam question our research is trying to answer is predicting which basketball team will win a game. The novelty of our approach is building a predictor based on the player types teams field for a game. Therefore, we aim to analyse the hypothesis: “*The winner of a game can be predicting by using mainly*^1^ *player's stereotypes*”*.*

### 1.3 Structure of our research

Following the Introduction, Section 2 discusses existing research on machine learning in sports, with a specific focus on basketball, how the unsupervised (clustering) and supervised machine learning techniques we employed are applied in similar settings and problems. Building on that, Section 3 depicts our solution design and key principles, Section 4 explains our results and our conclusions are presented in Section 5.

## 2 Literature review

In this section, we initially provide a summary of the relevant prior research on machine learning in sports by focusing on research on team performance and prediction, classification of players, and the dynamics of game and intra-game sequences.

### 2.1 Research on team performance and game outcomes

Kuehn (2017) uses a probabilistic model to identify how players complement or inhibit each other and models complementary skill sets. In this study, Kuehn models each possession within a game as an event tree, capturing potential outcomes. The probability of each node in the event tree is driven by another model – the individual player model. This model estimates, for every player in the line-ups of both teams, the probability for each player-event pair in the event tree. To estimate these probabilities, Kuehn uses player's propensities calculated from historical play-by-play data for each player. Feeding this into the event tree, the expected points per possession are calculated. By doing so, this study aims to capture the interaction effects among teammates as well as the effect from the opposition's line-up when attempting to predict how well a particular line-up can fare against another. In a similar study, Arcidiacono et al. (2017) employ a probabilistic model to estimate the productivity spillover of players and show that team performance is significantly impacted by players' ability to help their teammates to score.

Another study that focuses on player interactions is performed by Lutz (2012). In this study, Lutz identifies ten clusters of NBA players, using a combination of traditional and advanced box score statistics and a subset of shot selection data for players. Analysing the relationship between the existence of each cluster and team success via T-tests for each cluster, Lutz then expands on that, also analysing the interaction effects of clusters by analysing two cluster and three cluster combinations against team success. The “aggressive bigs” cluster is identified as having the lowest percentage of its players in winning teams, and also the “perimeter scorers” cluster is usually incompatible with other clusters. We consider this study to be one of the rare studies trying to model the positive and negative synergies between different playing styles.

However, the majority of team performance prediction studies use team-level statistics (e.g., points per game, rebounds per game aggregated at team level). Beckler et al. (2013), for example, aggregate NBA player's game-by-game statistics at team level across ’91–92 to ’96–97 seasons. Using linear regression, logistic regression, support vector machines, and artificial neural networks separately, they achieve an overall 70% accuracy with linear regression. Interestingly, they find that ANNs perform the worst, with only 65% overall accuracy. They also find that all their models perform relatively poorly in some seasons and argue that these seasons are inherently more ambiguous. Cao (2012) also uses team-level inputs for NBA games, employing logistic regression, Naïve Bayes, SVM, and ANNs separately, achieving prediction accuracy levels ranging from 66% (Naïve Bayes) to 68% (logistic regression), relatively on par with other research and human experts. Loeffelholz et al. (2009) employ multiple neural network types, fusing different models through a Bayesian Model to achieve an overall prediction accuracy of ∼72%, compared favourably to human expert scores of ∼69%.

Hu and Zidek (2004) adopt a novel approach in prediction with team-level inputs; they use weighted likelihood method to predict the outcome of the NBA Final Series of ’96–97 season. They use the in-season match history of the two teams to estimate the likelihood of the teams winning each game of the finals. Their models all estimate a Bulls championship, with probabilities ranging between 61% and 71% (actual outcome was a Bulls victory).

Yang and Lu (2012) employ support vector machines in an NBA setting. They identify the 16 playoff teams across ten seasons and analyse the games in every season between the playoff teams (i.e., 16 teams and 15 opponents for a single year yields 240 data points, this totals to 2400 across 10 years). They use SVM to predict the outcomes of the games played, using team-level inputs and achieve a 55% prediction accuracy.

Match outcome prediction, obviously, is not limited with basketball. Researchers have been trying to employ similar techniques in other settings to predict outcomes and understand the dynamics of team performances. Blaikie et al. (2011) combine team-level game statistics with data points external to the game (e.g., stadium attendance, team rankings etc.) as inputs to ANNs for National Football League (NFL) and College Football (NCAA). To test which variables have the best predictive capabilities, they use five different input spaces: using only in-game statistics, using only efficiency metrics, applying data reduction methods (principal component analysis and linear regression combinatorial optimization), and also using the full set of 46 input variables they gather. They analyse the results season by season and find that their models rank relatively favourably against experts for NFL but not for College Football. Another study on NFL teams uses team rankings published by The New York Times and using probit regressions to estimate the outcomes of the games (Boulier, 2003). Bouiler shows that this model can compete with human experts with prediction yet fail to perform better than a naïve predictor (i.e., home team wins).

David et al. (2011) employs a committee of committees approach to merge outputs from numerous committees, each comprising 50 ANN models predicting scoring margins of NFL games. They achieve accuracy scores ranging between ∼62% and ∼68% across three seasons, comparing favourably to other NFL predictors.

Flitman (2006) studies Australian Football League across three seasons to create ANN models via genetic algorithms, also assigning a probability score to outputs, showing how confident the model is in its prediction. Using this confidence score, Flitman calculates tipping scores for this model, penalising incorrect predictions with high confidence and rewarding little for correct prediction with low confidence. This allows, to a certain extent, the ability to distinguish the models that have, coincidentally, achieved relatively higher accuracy scores within the data set.

Leung and Joseph (2014) adopt a similar approach used by Hu and Zidek (2004) but they employ it in the context of football. They also use a clustering analysis to identify teams with similar playing styles and use the outcomes of games played between similar teams for prediction. In our view, this is a successful way to compensate for the relatively smaller set of matches played between the same teams compared to basketball.

Huang and Chang (2010) and Arabzad et al. (2014) both use ANN to predict football team's performances. Tax and Joustra (2015) also employ ANNs in football setting, alongside various other predictors and state that Naïve Bayes and ANNs perform other predictors they employ (e.g., Random Forest, decision trees etc.).

Existing research on the field does not only focus on predicting the outcomes. Another area of focus is identifying the similarities of players. Ultimately, this is done with the aim of optimising team configurations but still this research employ different approaches. Miller et al. (2014) perform a spatial analysis of shot selection data of NBA players by using spatial decomposition. They show that players have different offensive propensities in their shot selections and that the shot selection or shooting efficiency is not always invariant to the x-coordinate of the court (i.e., symmetrical between right wing and left wing). Rangel et al. (2019) assess the versatility of basketball players by using the individual box score data to predict the likelihood of that player manning each of the 5 traditional positions. They find that in the Brazilian Basketball League, the ratio of players not fitting into a single position is increasing rapidly, cementing our view that traditional positions in basketball are no longer adequate in capturing the play styles. Zhang et al. (2018) employ clustering analysis to identify similar players in the NBA, agnostic of traditional positions. They also use anthropometric properties and experience. They identify five clusters yet are unable to establish any connection between team performance and the team configuration based on the clusters they identify. We agree with their view on the need for additional labels/classifications for players.

Using a different approach to identify similar basketball players in the NBA, Piette et al. (2011) adopt network modelling approach and utilise play-by-play data spanning four seasons. They create a network of players where nodes (players) share an edge if they have played together in a five-man line-up. The weight of the edge is identified by the offensive, defensive, and total efficiencies of the line-ups the nodes (players) shared. Fewell et al. (2012) also adopt a strategic network approach to basketball and use network related measures (e.g., centrality) to classify player's roles and importance, however they lack the efficiency angle introduced by Piette et al. (2011). Again, in the context of NBA, Hore and Bhattacharya (2018) utilise SVM to identify player similarities but using a time series view. They use support vector machines to identify similar career trajectories of players across seasons and assess the likelihood of a given player to survive the first five years in the NBA. Dežman et al. (2001) use expert-defined criteria, instead of in-game statistics, to identify the positions of a sample of basketball players from the Croatian 1st League. They find that delineating the boundaries among small forward, shooting guard, and power forward positions is not practical, further strengthening our stance on the position fluidity.

### 2.2 Our contribution to the existing literature

Building on the existing literature, we aim to capture the synergies among players to help with our predictions. Our approach to achieve this differs from the player level analysis by Kuehn or the team level analysis by Leung and Joseph. We also agree with Lames and McGarry (2007) on their assertion that the aggregation of player level statistics to team level do not completely reflect the underlying traits researchers are trying to measure.

Therefore, we use clustering analysis to identify players with similar playing styles and use these as inputs instead of assessing everyone as a separate data point. This concept of prototype heuristics is a well-known human cognitive function, enabling us to quickly analyse complex systems (Kahneman and Frederick, 2012). These player styles help us capture cases where players classified as sharing the same traditional position (e.g., point guard, small forward etc.) and of similar calibre but would have different strengths and weaknesses and therefore their contribution to winning would vary significantly in the exact same situation.

We recognise that we lose precision in our model as none of the cluster members are exactly alike and yet our prediction models treat them the same. However, we consider this an acceptable trade off because using player types instead of the actual players gives us a richer data set on how similar players fare together with/against each other. This is mainly because there are a limited number of games where any two players play against each other but with the abstraction provided by clustering, we can train prediction models using all instances where similar player types played against each other.

## 3 Research method

### 3.1 Overview

We use a two-fold approach in our prediction engine: we first run a clustering method to identify the player types and then use these player types in each team to predict the game's outcome.

#### 3.1.1 Clustering

For our clustering approach, we build on the research of Lutz (2012), Miller et al. (2014) and Rangel et al. (2019). Lutz (2012) focuses on identifying players with similar styles through a data set that lacks efficiency stats (e.g., missed shots, advanced stats such as true shooting percentage etc.). Whilst Rangel et al. (2019) use traditional box score metrics to capture efficiency, the data they employ for clustering lacks the means to identify different styles, whereby Miller et al. (2014) point out in their research that the spatial distribution of players’ shot selection carries significant information on the characteristics of their playing styles. We therefore employ a data set that include combinations of traditional box score statistics, advanced efficiency metrics, and shot placement data to capture both playing styles and the efficiency of each player in the cluster memberships.^2^

We also recognise the fact that players can evolve through their careers: gaining new skills and losing the athletic abilities they possessed in their youth. To capture this evolution, we run our clustering algorithms once for every season and reclassify the players, using the classifications of the previous season for the predictions of the next season. We recognise that this approach introduces a limitation: we are unable to capture any new skills or playing styles developed over the summer and training camps. We are planning to address this limitation in our future work through a staggered clustering analysis that is run multiple times across the season.

In addition to a player's evolution over their career, we also acknowledge the fact that some players can adapt to their ecosystem and change their playing styles (Travassos et al., 2013). This phenomenon is known as phenotype plasticity in ecology literature (Werner and Peacor, 2003). This presents another potential risk in our approach: some players are highly versatile and can adapt their playing styles in response to the configuration of their team and the opponents. We mitigate the risk of classifying these versatile players strictly within a single cluster by using two clustering algorithms in parallel: k-means and c-means. We expect that the highly versatile players that are shoehorned into a single cluster in k-means will be better reflected in the fuzzy clustering outputs and this information will improve the prediction of games involving such players.

We are also cognizant of the distorting effects of Euclidean distance in high-dimensional data sets (Aggarwal et al., 2001). Therefore, we experiment with Manhattan, Mahalanobis, cosine, and Chebyshev distance metrics to account for the high dimensionality of our datasets. However, we find no significant differences on the predictors trained with clusters using these distance metrics (Euclidean distance and cosine distance being the best performers by ∼3–4% in overall prediction accuracy), we opt to report only Euclidean distance metric results.^3^

#### 3.1.2 Artificial neural networks

Bunker and Thabtah (2019) and Herold et al. (2019) state in their respective reviews of the existing research on machine learning in sports, artificial neural networks are one of the most widely used prediction methods. We opt to employ ANNs as our prediction algorithm, following the approaches laid out by Beckler et al. (2013), Cao (2012), Loeffelholz et al. (2009), and Yang and Lu (2012). We employ genetic algorithms to optimise the hyperparameters^4^ of the ANNs^5^ we train.

Despite our earlier criticism on aggregating player statistics to team level and using these for prediction, we agree that some external events have impacts at team level and therefore need to be factored into predictive models. Therefore, we include “the number of rest days” (Bunker and Thabtah, 2019), home & away teams to capture home team advantage (Nevill and Holder, 1999), and “the calendar month in which the game is played” to account for any seasonality effects (e.g., tanking) (Price et al., 2010; Soebbing and Humphreys, 2013; Walters and Williams, 2012) to our predictors’ input space.

### 3.2 Data gathering and preparation

The data we utilise for our research is acquired from public sources. We use seasonal player statistics for NBA players and box score data sourced from www.basketballreference.com from ’12–13 to ’17–18 seasons. For the same period, we gathered shot selection data for each NBA player from www.nbaminer.com website. We use regular season data and leave out playoff games to ensure a balanced data set for all teams.

#### 3.2.1 Data structure

The data we gather on player performance includes three key elements:

• Traditional basketball box score data, including but not limited with, points scored per game, rebounds per game, assists per game etc.;
• Advanced efficiency metrics, including true shooting percentage, effective field goal percentage, rebound rate, assist rate etc.; and
• Spatial distribution of player's shots across the 14 sections of the court (e.g., restricted area, in the paint but non-restricted area, right corner 3-point area, left corner 3-point area etc.).

A complete list of our clustering attributes and the definitions of each is depicted in Table 1.

**Table 1:** Player attributes used for clustering.

Abbreviation	Attribute definition
MP	Minutes Played Per Game
GS%	Percentage of games player was part of the starting five
PS/G	Points scored per game
FG	Field Goals Per Game
FGA	Field Goal Attempts Per Game
FG%	Field Goal Percentage
2P	2-Point Field Goals Per Game
2PA	2-Point Field Goal Attempts Per Game
2P%	FG% on 2-Pt FGAs.
3P	3-Point Field Goals Per Game
3PA	3-Point Field Goal Attempts Per Game
3P%	FG% on 3-Pt FGAs.
FT	Free Throws Per Game
FTA	Free Throw Attempts Per Game
FT%	Free Throw Percentage
ORB	Offensive Rebounds Per Game
DRB	Defensive Rebounds Per Game
TRB	Total Rebounds Per Game
AST	Assists Per Game
STL	Steals Per Game
BLK	Blocks Per Game
TOV	Turnovers Per Game
PER	Player Efficiency Rating. A measure of per-minute production standardised such that the league average is 15
TS%	True Shooting Percentage. A measure of shooting efficiency that takes into account 2-point field goals, 3-point field goals, and free throws
eFG%	Effective Field Goal Percentage. This statistic adjusts for the fact that a 3-point field goal is worth one more point than a 2-point field goal
3PAr	3-Point Attempt Rate. Percentage of FG Attempts from 3-Point Range
FTr	Free Throw Attempt Rate. Number of FT Attempts Per FG Attempt
ORB%	Offensive Rebound Percentage. An estimate of the percentage of available offensive rebounds a player grabbed while he was on the floor
DRB%	Defensive Rebound Percentage. An estimate of the percentage of available defensive rebounds a player grabbed while he was on the floor
TRB%	Total Rebound Percentage. An estimate of the percentage of available rebounds a player grabbed while he was on the floor
AST%	Assist Percentage. An estimate of the percentage of teammate field goals a player assisted while he was on the floor
USG%	Usage Percentage. An estimate of the percentage of team plays used by a player while he was on the floor
STL%	Steal Percentage. An estimate of the percentage of opponent possessions that end with a steal by the player while he was on the floor
BLK%	Block Percentage. An estimate of the percentage of opponent two-point field goal attempts blocked by the player while he was on the floor
TOV%	Turnover Percentage. An estimate of turnovers committed per 100 plays
Above the Break 3-Usage	Percentage of player's shots taken from the 3-point area above the break
Mid-Range Usage	Percentage of player's shots taken from the point zone but outside the paint
In the Paint (Non-RA)- Usage	Percentage of player's shots taken from within the paint but outside of RA
Restricted Area-Usage	Percentage of player's shots taken from within the RA (including dunks and layups)
Right Corner 3-Usage	Percentage of player's shots taken from the 3-point area in the right corner
Left Corner 3-Usage	Percentage of player's shots taken from the 3-point area in the left corner
Backcourt- Usage	Percentage of player's shots taken from the backcourt
Above the Break 3%	Making percentage of player's shots from that specific area
Mid-Range %	Making percentage of player's shots from that specific area
In the Paint (Non-RA) %	Making percentage of player's shots from that specific area
Restricted Area %	Making percentage of player's shots from that specific area
Right Corner 3%	Making percentage of player's shots from that specific area
Left Corner 3%	Making percentage of player's shots from that specific area
Backcourt %	Making percentage of player's shots from that specific area

We then standardise our data to mitigate the nominal attributes and apply dimensionality reduction to mitigate the hyper dimensionality effects as identified by Aggarwal et al. (2001). We apply Principal Component Analysis with a cut off defined at 90% of the cumulative variance explained and identify 15 components. We use the PCA outputs, in parallel to the initial datasets during the clustering phase, effectively running each clustering configuration twice for each.

### 3.3 Clustering

#### 3.3.1 Player clustering using k-means algorithm

The first clustering algorithm we implement is k-means, with Euclidean distance as the distance metric, using the Python libraries by Pedregosa et al. (2015). Instead of committing to a small set of potential values for *k* and then assessing their validity retrospectively, we adopt a style more akin to brute force. We run our k-means algorithm for all values of *k* between 5 and 35. To account for the potential impact of random centroid seeds on the cluster robustness, we run each clustering instance of the algorithm 10 times, with different random seeds, selecting the best performing clustering output, using Python’s scikit learn library. For each of these clustering outputs, we estimate the cluster validity using Silhouette value and Calinski-Harabasz pseudo-F index for each run of the algorithm (Chan et al., 2012). Using these two validity indices, we identify best performing *k* values as k = 25 and k = 26. We also recognise that Bezdek and Bezdek (1981) shows that no cluster validity measure is able to capture the correct number of underlying clusters in every case. Therefore, we also randomly sample other *k* values to use as inputs for our predictors. We find that as the *k* value deviates from the 25–26 range in either direction, the prediction success drops.^6^ We discuss, in more detail, the characteristics and prominent members of these 26 clusters in Section 4.2.

#### 3.3.2 Player clustering using c-means algorithm

The ability to identify a player's propensities across numerous playing styles is a valuable capability since we assume that phenotypic plasticity is not a rare trait in professional basketball players. We use the Python libraries created by Virtanen et al. (2020) to implement the c-means clustering. Our approach to implementation is virtually identical to our approach for k-means, with two key differences:

• In addition to Euclidean distances, we also use cosine, Manhattan, Mahalanobis, and Chebyshev distance metrics for our c-means implementation. Aggarwal et al. (2001) prove that, in high dimensional data sets Euclidean distance-based similarity measures become ill-defined as the distances between a target point's nearest neighbours and farthest neighbours tend to converge.
• Instead of the Silhouette value and Calinski-Harabasz pseudo-F index to estimate clustering validity, we use the partitioning coefficient, a validity score built on the distribution of cluster memberships (Bezdek and Bezdek, 1981).

Our clustering outputs show that cosine distance outperforms other distance metrics for *c-means*.

### 3.4 Prediction

To create the input space for predictors, we map the cluster memberships generated to team rosters, on a game-by-game basis. We calculate the total number of minutes played by players belonging to each cluster for the home and away teams separately. If more than one player in a team is assigned to the same cluster, their minutes are aggregated to calculate how this cluster is presented for this team in that game.

Please note that as discussed in Section 3.1, we also include the following team-level attributes that are external to the game:

- Number of days between the game date and the last game played by home and away teams;
- Dummy variables for the month of the season; and
- Winning percentage of each team up to the game date in that season.

Flitman (2006), Tax and Joustra (2015), Arabzad et al. (2014) have all successfully employed genetic algorithms to improve their prediction models in sports analytics. We have built our implementation of genetic algorithm ANNs (GA ANN) by modifying the DeepEvolve framework (Liphardt et al., 2019). The ANNs are built on TensorFlow using Keras, with the genome of each model coding data on:

• Number of layers between two and five;
• Number of neurons in each layer randomly selected from a list of (4, 6, 8, 12, 16, 24, 32, 48, 64, 96, 128, 256);
• Activation function is selected randomly from (rectified linear unit function, exponential linear unit function, softplus, softmax, sigmoid activation function, hard sigmoid activation function, hyperbolic tangent activation, and identity function);
• Optimization function is selected randomly from (RMSProp optimizer, Adaptive moment estimator, Stochastic gradient descent optimizer, Adagrad optimizer, Adadelta optimizer, Adamax optimizer (Kingma and Ba, 2015), Nesterov Adam optimizer; and
• Learning rate is randomly selected from (0.1, 0.01, 0.001, 0.0001).

For each input version (i.e., each clustering output we used to train predictors), we train 300 generations, beginning with 500 individuals in a generation. For each generation, we retain the fittest 25% of that generation, as well as randomly selecting 10% of the rest of the individuals. Following breeding, we employ a 10% probability for each gene to undergo a random mutation.

We provide the complete ANN input space for this example (Orlando Magic @ Indiana Pacers game on 29/10/2013) in Table 2.

**Table 2:** Complete input space for ANN predictors used for the Orlando Magic @ Indiana Pacers game on 29/10/2013 using the 25-cluster configuration produced by k-means.

Input Attribute	Value	Input Attribute	Value
Home team rest day	0	Away 20	0
Away team rest day	0	Away 21	0.058091
Home WP^1^	N/A	Away 22	0
Away WP^2^	N/A	Away 23	0.128631
October	1	Away 24	0
November	0	Home 0	0
December	0	Home 1	0
January	0	Home 2	0
February	0	Home 3	0
March	0	Home 4	0.062241
April	0	Home 5	0.120332
Away 0	0	Home 6	0
Away 1	0	Home 7	0
Away 2	0.107884	Home 8	0
Away 3	0	Home 9	0.149378
Away 4	0.128631	Home 10	0.149378
Away 5	0.26556	Home 11	0
Away 6	0	Home 12	0.037344
Away 7	0	Home 13	0
Away 8	0	Home 14	0
Away 9	0	Home 15	0
Away 10	0	Home 16	0
Away 11	0.037344	Home 17	0
Away 12	0	Home 18	0
Away 13	0.095436	Home 19	0.153527
Away 14	0.078838	Home 20	0.240664
Away 15	0	Home 21	0.087137
Away 16	0.099585	Home 22	0
Away 17	0	Home 23	0
Away 18	0	Home 24	0
Away 19	0		

1 Please note that due to this game being the first game of the season for both teams, this data point is null.

2 Please note that due to this game being the first game of the season for both teams, this data point is null.

We use a 75%–25% random training-test split, totalling to 4612 game scores for training 1,538 game scores for testing. We note that our approach ignores the temporal dimension when doing the test split. This is for parsimony reasons. We maintain a consistent universe of clusters (with memberships changing every year) and assume that the underlying positive and/or negative synergies between clusters will not significantly change over time. We recognise this as a limitation of our current study and plan to pursue this in further research.

## 4 Analysis of results

In this section, we discuss the results of our analysis in two phases, starting with the interpretation of a sample of the clustering outputs and then discussing the performance of our prediction models in detail.

### 4.1 Clustering analysis

Using Silhouette Score and Calinski-Harabasz pseudo-F index, we analyse the genesis of k-means algorithms.^7^ For k-means we see that the highest performing clustering outputs are achieved by 25 and 26 clusters. It is worth noting that, as stated earlier, we have only used Euclidean distances during our implementation of the *k-means* algorithm.

Our c-means algorithms yield different outcomes. 10 cluster configurations using cosine distances outperform all other configurations *c*-means configurations. Existing research shows that in clustering with high-dimensional data, cosine distance can outperform Euclidean distance (Sahu and Mohan, 2015). Using cosine metric generated outputs leads to an interesting trade off: players with similar tendencies will be more likely clustered even though their nominal output may be different. With cosine distance metric sacrificing the difference between quantities per attribute for the sake of identifying vectoral similarities, we feel that clusters that may be overlooked by k-means could be captured. The other distance metrics perform significantly worse in our c-means implementation, across all ranges of cluster numbers.

### 4.2 Analysis of an example clustering output

In this subsection we discuss, in more detail, one of the highest performing clustering configurations. This clustering result was achieved using the *k*-means algorithm and *k* value of 26. This configuration has performed strongly not only in validity indices but also in training predictors with high accuracy (∼76%, we discuss prediction accuracy of our models in Section 4.3 – Game outcome predictions). We want to emphasise the fact that the cluster structure we shall dissect in this section is but one of the high performing intermediate products. It is the one that has led to the training of the predicting model with one of the highest accuracy levels we achieved but by no means this guarantees that it is the best clustering structure explaining the dynamics and player roles. In Table 3, we discuss the 20 most prominent clusters (out of 26 clusters)^8^ in terms of minutes played, usage rates, and their impacts on key statistical attributes alongside examples of prominent members for each cluster. Please note that whilst each cluster is labelled during our analysis, some inevitably contain fringe players in terms of minutes played and statistical contributions, so we only focus on the more interesting clusters in this example.

**Table 3:** In-depth analysis of a clustering output example.

Label	Comments and characteristics	Some of the notable members
Elite bigs	This cluster, as the name suggests, is composed of efficient and multi-faceted players who can rebound, assist, and score in the paint and midrange quite efficiently, despite their incredibly high usage rates.	’14–15, ’15–16, ’16–17, and ’17–18 seasons of Anthony Davis ’13–14 and ’14–15 seasons of Tim Duncan
Offensive juggernauts	These players have no offensive weak spots, despite their high usage rate, they distribute their shots relatively well among midrange, painted area, and 3 pointers. They also boast healthy assist rates, placing them as the focal point of their team's offence.	’14–15 and ’15–16 seasons of Kobe Bryant ’13–14, ’14–15, and ’15–16 seasons of Kyrie Irving
Swiss army knives	These players are the embodiment of versatility. Their shot selection is similar to “All around scorers”, albeit with less midrange usages and more 3 pointers. Yet they contribute more in rebounds, assists, steals, and blocks, leading to a high efficiency rating.	’13–14, ’14–15, ’15–16, ’16–17, and ’17–18 seasons of Kevin Durant ’13–14, ’14–15, ’15–16, ’16–17, and ’17–18 seasons of LeBron James ’16–17 season for John Wall
Bruisers	These players live and die in the restricted area. They have incredibly high rebounding and block rates, taking an astounding 74% of their shots from the restricted area. They also boast remarkably high player efficiency ratings and shooting efficiency.	’13–14, ’14–15, ’15–16, ’16–17, and ’17–18 seasons of Dwight Howard ’14–15, ’15–16, and ’16–17 seasons of Hassan Whiteside
Offensive focal point	These players have a unique blend of high usage rate, assist rates, and painted area usage. They prefer to shoot relatively less 3 pointers compared to similar groups with similar assist and/or usage statistics. They also have low rebound rates and block rates, further suggesting that their key duty is setting up their teammates to score and scoring predominantly via lay ups or post up plays.	’13–14, ’14–15 (for both Boston Celtics and Dallas Mavericks), ’15–16 (both for Sacramento Kings and Chicago Bulls), ’16–17, and ’17–18 seasons of Rajon Rondo ’13–14, ’14–15, ’15–16, and ’17–18 seasons for John Wall
Jack of all trades	This cluster is a slightly different version of the Swiss Army Knives, they still boast large usage rates, efficiency figures across the board but with lower 3 pointers and assist rates and higher rebound and painted area usage.	’13–14, ’14–15, ’15–16, ’16–17, and ’17–18 seasons of Dirk Nowitzki ’14–15, ’15–16, ’16–17, and ’17–18 seasons of Al Horford
Combo wings	Combining a high usage rate and a large portion of shots from the 3-point territory, these players also have a relatively high level of assist rates. This suggests that they span the traditional point guard and shooting guard duties as their natural modus operandi.	’13–14, ’14–15, ’15–16, and ’17–18 seasons of Manu Ginobili ’13–14, ’14–15, ’15–16, and ’16–17 seasons of J.J. Barea
Stretch bigs	These players take more than 50% of their shots from the 3-point territory and yet still manage a 25% restricted area usage. They deliberately prioritise high-value shots, have relatively high rebounding rates, efficient shooting, and low assist rates. They are the front court players of the new era, foregoing low value post up play for the sake of high value 3 pointers and space created for their teammates.	’14–15 season of Ryan Anderson ’14–15, ’15–16, and ’16–17 seasons of Nikola Mirotic
Slashers	These players make their offensive impact from inside the 3-point line, shooting more than 70% of their shots from midrange or restricted area. Despite their relatively lower shooting efficiency compared to classes discussed above, they maintain a high usage rate and a healthy assist rate.	’13–14 and 15′16 seasons of Kyle Lowry ’15–16 season of Dwayne Wade
Playmakers	These players display a fair distribution of their shots across the 3-point area, midrange, and painted area. They also have high usage rates, relatively high assist rates and limited defensive efficiency.	’13–14 season (both Denver Nuggets and Washington Wizards) of Andre Miller ’14–15, ’15–16 (for both Miami Heat and Memphis Grizzlies), and ’17–18 seasons of Mario Chalmers
Inefficient scorers	Key characteristics of this cluster are their relatively high rebounding rates, usages rates and significantly poor offensive efficiencies.	’14–15 and ’15–16 seasons of Josh Smith ’17–18 season of Dejounte Murray
3p capable bigs	These players have modest usage and high rebound rates and relatively high assist rates. Their offensive efficiency is higher than average with more than half of their shots coming from the restricted area. Despite their offensive efficiency and high 3-point shot accuracy, they utilise the 3-point shot less than many other clusters.	’14–15, ’16–17, and ’17–18 seasons of Ersan Ilyasova ’17–18 season (both Chicago Bulls and New Orleans Pelicans) of Nikola Mirotic
D&D (defend and distribute)	These players boast remarkably high assist and steal rates along with relatively high usage rates, yet they utilise 3-points infrequently and have low rebound rates and overall efficiency.	’14–15 season (both Washington Wizards and Sacramento Kings) of Andre Miller ’17–18 season (both Cleveland Cavaliers and Miami Heat) of Dwayne Wade
Snipers	These players utilise the 3-point shot significantly and with deadly efficiency. On average they also have mediocre assist, rebound, and steal rate but their defining quality is their 3-point shot.	’13–14, ’14–15, ’15–16, ’16–17, and ’17–18 seasons of Klay Thompson ’13–14, ’14–15, ’15–16, ’16–17, and ’17–18 seasons of J.J. Redick
2nd tier bigs	These players have an elite combination of block, steal, and rebound rates. Their offensive efficiency is respectable but not stunning and their usage rate is mediocre. On average, their shot selection is heavily biassed towards painted area and midrange.	’13–14, ’14–15, and ’15–16 seasons of Kevin Garnett ’14–15, and ’15–16 seasons of Roy Hibbert
Scorer bigs	This cluster is defined by their mediocre usage rate, high preference to shoot from the painted area, high offensive efficiency and modest rebound rates.	’13–14, ’14–15 (both New York Knicks and Dallas Mavericks), and ’15–16 seasons of Amar'e Stoudemire ’13–14, ’14–15, ’15–16, and ’16–17 seasons of David Lee
Brick-layers	With their high usage rate and incredibly inefficient offensive ratings, they are highly inefficient players who still take a lot of shots, hurting their teams in the process.	’14–15 season of Gary Harris ’13–14 season of Earl Watson
Pass first	This cluster is characterised by their high assist rates and low usage rates meaning they shoot less than average yet pass a lot more. Their primary role is setting up the offensive flow. They also have reasonably high steal rates.	’15–16 season of Jeff Teague ’15–16 season (both Minnesota Timberwolves and San Antonio Spurs) of Andre Miller
Clamps/shackles	These players are the defensive specialists. Their key attribute is the high steal and block rates. Their offensive usage, rebound, and assist rates are all lower than average, indicating a specialist role for defence.	’13–14 Metta World Peace ’13–14, ’14–15, ’15–16, ’16–17, and ’17–18 seasons of Tony Allen
Vanguards	This cluster is characterised by their incredibly low offensive usage rate, remarkably high rebound and block rates, efficient yet low volume offence, and heavy bias towards operating from within the restricted area.	’13–14, ’14–15, ’16–17, and ’17–18 seasons of Ian Mahimi ’13–14, ’14–15, ’16–17, and ’17–18 seasons of Omer Asik

We think it is worth discussing our clustering outputs by applying a temporal lens as well. Careful readers will have already noticed that some players have been assigned to different clusters in different seasons or even during the same season in different teams (this can only happen if a player is traded to another team or bought out by his team and then signed by another team in mid-season). We had also briefly alluded to this feature of our solution design in Section 3. One of the key points in our design is its ability to capture the evolution of a player and/or assess how he fits in a new environment.

### 4.3 Game outcome predictions

Whilst we have experimented with training predictors with ∼150 different clustering configurations, three models outperform their peers in terms of prediction accuracy. The predictor we train with the input spaces generated by *c-means* using *cosine* distance metric and a *n* value of 10 achieved a prediction accuracy of 76.52% over our validation set. This is followed by the predictors trained with the input spaces generated by k*-means* and a *k* values of 26 and 25, achieving prediction accuracies of 76.29% and 75.36% respectively. Table 4 provides a summary view of these results and how they compare against benchmarks of:

• A naïve predictor of ‘home team wins’ (i.e., 58% prediction accuracy);
• A simple predictor trained through the same GA ANN approach with inputs consisting of the rest days and the to-date win percentages of the teams in the season the game takes place (i.e., 72.02%); and
• Human subject matter experts who have prediction accuracies in the range of 65%–68%, based on Beckler et al. (2013), Loeffelholz et al. (2009), Miljković et al. (2010), and Boulier (2003).

**Table 4:** Breakdown of prediction accuracy by predictor and space.

Model ID	Clustering Algorithm	Distance metric	*k* value	GA ANN accuracy (%)	GA ANN predictor with a simple input space (%)	Naïve predictor (home team wins %)	Human experts (%)^1^
1	c-means	Cosine	10	76.52	72.02	58	∼65–68
2	k-means	Euclidean	26	76.29			
3	k-means	Euclidean	25	75.36			

1 Based on Beckler et al. (2013), Loeffelholz et al. (2009), Miljković et al. (2010), and Boulier (2003), we define a range of 65%–68% prediction accuracy for human experts.

As seen above, our highest prediction accuracy region overlaps with the high values of clustering validity indices, reinforcing our initial prediction that these indices are a good proxy of our models’ capability to explain the underlying groups.

We also recognise the fact that some teams' games are harder to predict by the nature of their performances whilst some are easier. For example, the Golden State Warriors, across our data set, has won 83% of their home games and 71% of their away games, making predictions of such teams' outcomes relatively easier. In a stark contrast, the outcomes of the games for some teams are more difficult to predict (e.g., Dallas Mavericks have won 55% of their home games and 42% of their away games in our dataset). Therefore, we present the precision, recall, and F1 metrics for these three models on a team basis in Table 5. We observe strong variance in these metrics across the teams (e.g., for Golden State Warriors’ home games, our model has 0.86 precision, 0.99 recall, and 0.91 F1 whereas for Dallas Mavericks, these metrics drop to 0.68, 0.74, and 0.71 respectively). We are planning to address this limitation in future work by implementing a risk weight to our predictions.

**Table 5:** Per-team breakdown of precision, recall, and F1 scores of top 3 (in terms of accuracy) models.

Team	*c-means* with *n* = 10			*k-means* with *k = 26*			*k-means* with *k = 25*		
	Precision	Recall	F1	Precision	Recall	F1	Precision	Recall	F1
ATL	0.72	0.87	0.79	0.77	0.81	0.79	0.74	0.84	0.79
BOS	0.73	0.89	0.80	0.75	0.83	0.79	0.73	0.87	0.79
BRK	0.75	0.56	0.64	0.73	0.53	0.61	0.67	0.67	0.67
CHI	0.66	0.77	0.71	0.70	0.74	0.72	0.68	0.83	0.75
CHO	0.71	0.79	0.75	0.73	0.78	0.76	0.70	0.88	0.78
CLE	0.79	0.92	0.85	0.79	0.88	0.83	0.80	0.92	0.86
DAL	0.68	0.74	0.71	0.74	0.67	0.70	0.69	0.77	0.73
DEN	0.70	0.70	0.70	0.71	0.64	0.67	0.68	0.77	0.72
DET	0.71	0.84	0.77	0.78	0.72	0.75	0.68	0.84	0.75
GSW	0.86	0.99	0.92	0.87	0.98	0.92	0.86	0.98	0.92
HOU	0.79	0.97	0.87	0.81	0.93	0.86	0.78	0.97	0.87
IND	0.79	0.91	0.85	0.80	0.82	0.81	0.79	0.89	0.84
LAC	0.78	0.96	0.86	0.83	0.92	0.87	0.80	0.94	0.87
LAL	0.65	0.49	0.56	0.62	0.37	0.47	0.63	0.53	0.58
MEM	0.72	0.89	0.80	0.74	0.82	0.78	0.71	0.85	0.78
MIA	0.74	0.90	0.81	0.76	0.81	0.78	0.78	0.86	0.82
MIL	0.70	0.76	0.73	0.72	0.73	0.73	0.69	0.78	0.73
MIN	0.74	0.72	0.73	0.73	0.72	0.73	0.70	0.76	0.73
NOP	0.72	0.63	0.67	0.72	0.64	0.68	0.66	0.79	0.72
NYK	0.70	0.75	0.73	0.72	0.62	0.67	0.71	0.61	0.66
OKC	0.79	0.88	0.83	0.81	0.84	0.83	0.80	0.92	0.86
ORL	0.63	0.60	0.62	0.61	0.56	0.58	0.59	0.72	0.65
PHI	0.72	0.61	0.66	0.70	0.41	0.52	0.71	0.72	0.71
PHO	0.62	0.70	0.66	0.68	0.66	0.67	0.63	0.71	0.67
POR	0.80	0.93	0.86	0.80	0.92	0.86	0.79	0.94	0.86
SAC	0.67	0.62	0.64	0.63	0.48	0.54	0.68	0.51	0.59
SAS	0.85	0.98	0.91	0.86	0.95	0.90	0.85	0.98	0.91
TOR	0.76	0.98	0.86	0.80	0.94	0.86	0.76	0.98	0.86
UTA	0.75	0.78	0.77	0.81	0.70	0.75	0.72	0.82	0.77
WAS	0.69	0.87	0.77	0.70	0.79	0.75	0.68	0.80	0.74

### 4.4 Robustness checks

**Figure 1:**
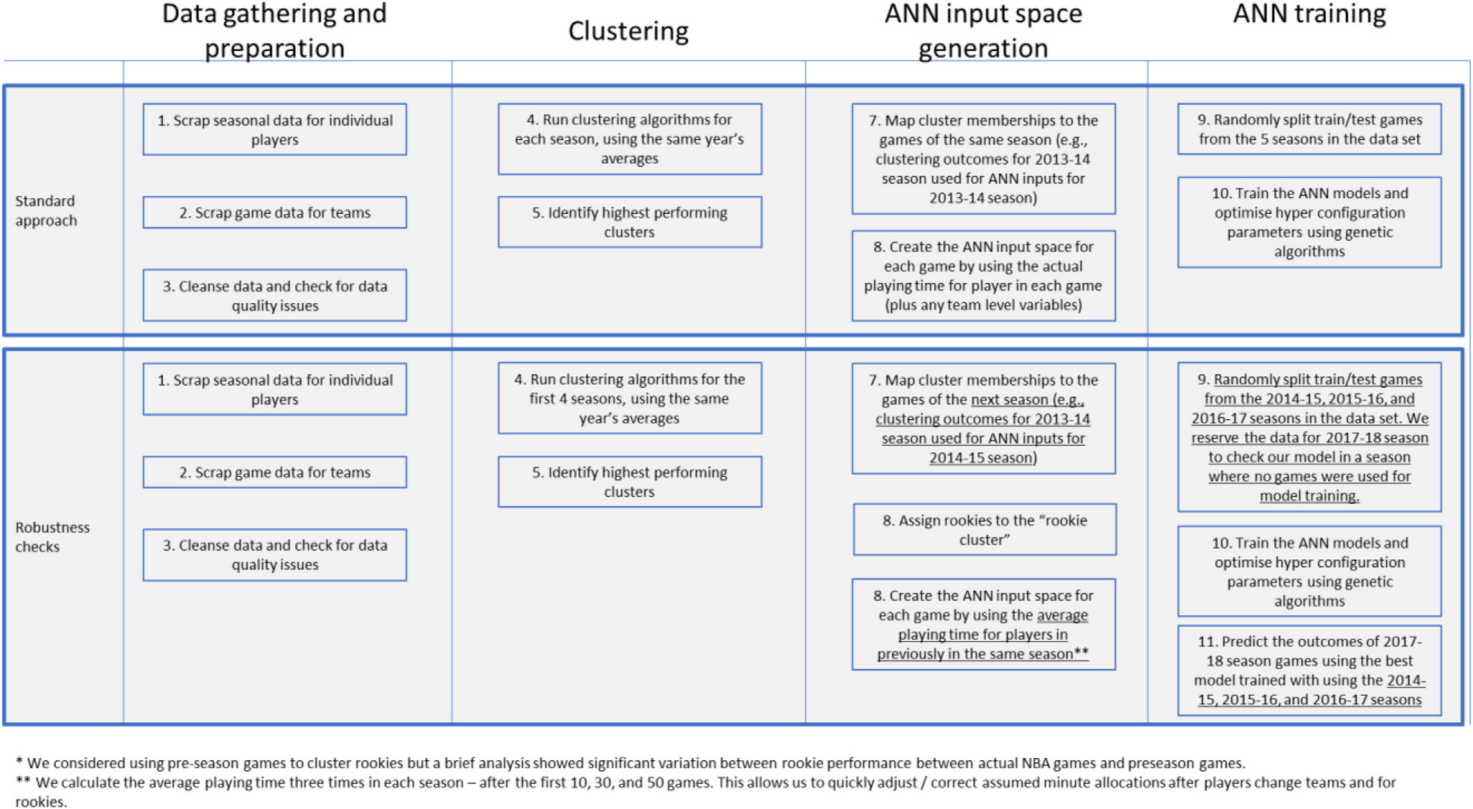
In-depth analysis of a clustering output example.

We recognise that our approach has certain limitations, some of which have been mentioned above. We perform some experiments with our approach to address some of these limitations and review whether our approach can still lead to models with high prediction accuracy. Table 6 below depicts these limitations, our robustness checks (where available) and their results. We also provide an overview of our end-to-end process from data gathering to ANN model training and how that end-to-end process changes in our robustness checks in Figure 1 below.

**Table 6:** Limitations and robustness checks applied.

Limitation	Robustness check applied	Results and implications
1. ANN inputs include data from the future (e.g., player stereotypes for the 2014-15 season are calculated with this season's averages. Output of this clustering is used to predict the same season's results)	We employ a staggered approach between clustering and ANN. To predict a game from *season x* we use *x-1 season's* clustering data. We use 2013–14 (first season in our data set) only to calculate cluster memberships. These are used to predict 2014-15 seasons' games. Clustering outcomes of 2014–15 is used for predictions of the 2015-16 season and the clustering outputs of 2015–16 to predict the 2016-17 season. We do not run clustering on the 2016-17 season and leave the 2017-18 games out of test/train data set. We use the 2017-18 season to independently assess the prediction powers of our model.	Our models trained with this approach achieve only slightly lower prediction accuracy scores (overall prediction accuracy of 75.03% compared to 76.3% of our initial approach). We think that the prediction accuracy of the robustness checks may be artificially inflated because of the smaller data set. This is because we expected a slightly larger drop in the prediction accuracy due to the information loss. When we run our new model for the 2017-18 season (which was not used in its training at all), we achieve a 70.65% prediction accuracy across that season. We think that these robustness check support our approach to use player stereotypes for prediction.
2. Our test/train split draws games randomly from all 5 seasons in our data set. We do not check the predictive capability of our models with a season that was not used for model training.		
3. Our ANN input space is calculated by using the actual minutes played by each player.	We retrain our models, using the average playing time for players in previously in the same season. We calculate the average playing time three times in each season – after the first 10, 30, and 50 games. This allows us to quickly adjust/correct assumed minute allocations after players change teams and for rookies.	We see no significant changes in the prediction accuracy of our models. We admit that the 10, 30, and 50 game thresholds to recalculate the minute allocations are chosen arbitrarily and the frequency can be adjusted for increased precision. We also appreciate that in-game events such as injuries or ejections can lead to significant discrepancies between the model inputs and actual minutes played. However, these can only be captured by in-game (e.g., possession by possession) prediction engines and this is beyond the scope of our research.
4. Our ANN input space uses team-level “Win %” statistics in addition to player stereotypes	We retrain our models without the “Win %” variables. These are proxies for the teams' power rankings, encapsulating all their wins and losses in a given season up until the game date. We remove this variable for both home and away teams and retrain our models.	As we expected, prediction accuracy of our models drops to: - 67.7% for the k-means with 26 clusters (from 76.29%); - 66.1% for the k-means with 25 clusters (from 75.36%); and −66.3 for the c-means with 10 clusters (from 76.52%). This shows that whilst the “Win %” contains significant information, our models can still compete with human experts without this variable, validating our approach.
5. For comparison, we apply the robustness check #2 to the simple predictor as defined in Section 4.3	We apply the robustness checks #2 to a model trained using only team level statistics (i.e., win percentages, rest days, and calendar month of the game variables). We reserve the last season's (i.e., 2017–18) data for assessing the prediction performance. We run predictions across the entire 2017-18 season and compare the prediction performance against our approach so that our models trained with the robustness checks are benchmarked against the simple predictor with the same robustness checks applied (i.e., not the 72.02% prediction accuracy achieved with the simple predictor without the robustness checks).	The models produced by team level variables achieve *67.34%* accuracy for the 2017-18 season. This is on par with our best performing model trained without these team level variables.
6. Our approach cannot capture any new skills or playing styles developed over the summer and training camps.	N/A	We are planning to address this limitation in our future work through a staggered clustering analysis that is run multiple times across the season.
7. Our approach is built on the assumption that the underlying positive and/or negative synergies between clusters will not significantly change over time.	N/A	This limitation is, to a certain extent, mitigated by using isolating the 2017-18 season from the test/training data set. However, we plan to further analyse this in further research by expanding our data set.
8. Our prediction algorithm does not discriminate between “safe to predict” vs “difficult to predict” games.	N/A	In our future research, we plan to add risk weightings for games and a committee of models to assess how confident each model is about their predictions for each game.

## 5 Conclusion

In this research, we aimed to find a reliable method to predict the outcome of NBA games. Our research helps build the theory on a significant gap in the literature by approaching the prediction problem from a complex system approach. Following a slightly similar approach to Lutz (2012), Oh et al. (2015), and Kuehn (2017), we identify different player stereotypes, completely agnostic of how players are labelled according to the 5 traditional positions in basketball. By identifying the different playing styles across the league, we aim to capture how well certain team configurations work by generating positive synergies, leading to more wins. We use clustering techniques to identify which clustering configuration would generate the best input space for predictors. When we add simple team level variables to our models, our prediction success increases to ∼71%^9^ through an entire season that was not used for model training at all. Our prediction results show that our principles and approach are sound: we manage to predict game outcomes more accurately than human experts and achieve level results with the highest performing models in similar research settings.

We think our research shows that by analysing player propensities and tendencies through protype heuristics, it is possible to predict the outcomes of basketball games with a reasonable degree of confidence.

## Declarations

### Author contribution statement

Cem Osken: Conceived and designed the experiments; Performed the experiments; Analyzed and interpreted the data; Contributed reagents, materials, analysis tools or data; Wrote the paper.

Ceylan Onay Sahin: Conceived and designed the experiments; Wrote the paper.

### Funding statement

This research did not receive any specific grant from funding agencies in the public, commercial, or not-for-profit sectors.

### Data availability statement

Data used is publicly available and cited as such.

### Declaration of interest's statement

The authors declare no competing interests.

### Additional information

No additional information is available for this paper.

## Footnotes

1 We also employ some team level (e.g., rest days for each team) and global (the month the game is played) inputs. However, our robustness checks show that even when only player types are used for prediction, we can train successful models.

2 Please see Table 1 for the complete list of attributes used for clustering.

3 The remaining results are available upon request.

4 We also experiment with SVMs for prediction. As expected, SVMs are ∼3 orders of magnitude quicker to train yet do perform poorly (on average, SVMs perform ∼4.2% poorer on prediction accuracy) compared to GA optimised ANNs. Detailed results are available upon request.

5 We also experiment with random grid search techniques but as expect these do not perform as well as the genetic algorithms so for the sake of parsimony, we only report the outputs of the genetic algorithm optimised ANNs. The results of random grid search optimisations are available upon request.

6 These results are available upon request.

7 For parsimony reasons, we omit the full results of the Silhouette coefficient and Calinski-Harabaz Index scores. However, for both validity indices, the elbow is reached in the 24–29 cluster region, with Silhouette score ranges of 0.85328 to 0.80045 and Calinski-Harabaz Index of 7387.72 to 3372.67.

8 The cluster labels not explained in the table are: ‘passer-snipers’, ‘point forwards’, ‘3&D’, ‘fringe bigs’, ‘mid-tier scorers’, and ‘3-point specialists’.

9 When we calculate our prediction success across our entire data set, including the games used for model training, we achieve ∼76% prediction accuracy.
